# Are Auditory Percepts Determined by Experience?

**DOI:** 10.1371/journal.pone.0063728

**Published:** 2013-05-07

**Authors:** Brian B. Monson, Shui’Er Han, Dale Purves

**Affiliations:** 1 Neuroscience and Behavioral Disorders Program, Duke-NUS Graduate Medical School, Singapore; 2 Department of Neurobiology, Duke University Medical Center, Durham, North Carolina, United States of America; 3 Center for Cognitive Neuroscience, Duke University, Durham, North Carolina, United States of America; University of Salamanca- Institute for Neuroscience of Castille and Leon and Medical School, Spain

## Abstract

Audition–what listeners hear–is generally studied in terms of the physical properties of sound stimuli and physiological properties of the auditory system. Based on recent work in vision, we here consider an alternative perspective that sensory percepts are based on past experience. In this framework, basic auditory qualities (e.g., loudness and pitch) are based on the frequency of occurrence of stimulus patterns in natural acoustic stimuli. To explore this concept of audition, we examined five well-documented psychophysical functions. The frequency of occurrence of acoustic patterns in a database of natural sound stimuli (speech) predicts some qualitative aspects of these functions, but with substantial quantitative discrepancies. This approach may offer a rationale for auditory phenomena that are difficult to explain in terms of the physical attributes of the stimuli as such.

## Introduction

A puzzling observation in audition is that the psychophysical functions of loudness and pitch are not proportional to measured sound pressure levels and sound wave frequencies in acoustic stimuli [1]–[3]. Many studies have sought to explain this fact using the transfer functions imposed by the external, middle and inner ear and/or the consequent firing rates of auditory nerve fibers, under the assumption that auditory percepts are elicited by filtering, analyzing, and representing stimulus intensity and frequency in the higher auditory path and auditory cortex [1], [4].

Such studies don’t often address how experience contributes to auditory perception, although considerable evidence indicates that experience with natural stimuli influences, or even determines, how the auditory or other sensory systems lead to perceptions of the environment [5], [6]. The importance of empirical information follows from the fact that agents must behave in response to sensory stimuli that, due to the nature of biological sensors, conflate their underlying physical sources (in vision, this is known as the inverse optics problem). For example, a two-dimensional retinal image can arise from many possible three-dimensional sources. A plausible way to cope with the inherently uncertain link between stimuli and their sources is to depend on accumulated experience based on trial and error behavioral responses over evolutionary and individual time, retaining pre-neural mechanisms and neural circuitry that led to behavioral success [7], [8]. The role of experience has figured importantly in various other theories of human perception, including Helmholtz’s original idea of unconscious inferences, inferences based on Bayesian decision theory, and inferences derived from gestalt principles [9]–[11].

If experience with natural stimuli is the main source of information that determines perception, a reasonable expectation is that percepts should be predicted in part by the frequency of occurrence (i.e., the probability distribution) of natural stimulus patterns. Since prediction of ordinal psychophysical functions (e.g., lightness, loudness, etc.) entails a monotonically increasing function, the cumulative distribution function (CDF), obtained directly from the probability distribution, may be useful in predicting perceptual functions. The CDF is a percentile scale that indicates the percentage of stimuli that are lower by some physical measure (e.g., luminance or acoustic intensity) than a particular stimulus, and the percentage of stimuli that are higher. Thus any stimulus is ranked by its relationship to all other stimuli on this scale. Since the slope of the CDF is proportional to the frequency of occurrence, the CDF has the further advantage of providing greater discriminative information (resolution) for those stimuli that occur most often. The CDF has similarly been proposed as a scale that predicts intensity-response functions of sensory neurons that follow efficient coding principles of information maximization [12], [13]. Recent studies in vision have used the CDF of stimulus patterns from the visual environment to accurately predict many basic human psychophysical functions [7], [8], defining the “empirical ranking theory”. (Here and throughout the text we use to the term “empirical” to refer to this theoretical framework.) Here we propose that audition may also operate in this fashion.

In an empirical conception of audition, perceived qualities such as loudness and pitch do not reflect sound pressure and frequency as such, but rather the relationship of a given pattern of sound pressure and frequency to all similar patterns experienced over phylogeny and ontogeny. To test the merits of this interpretation of audition, we asked whether the CDFs of acoustic patterns in a database of commonly experienced natural sounds tracks five well-documented human psychophysical loudness and pitch functions. We analyzed a database of speech as a representative subset of natural sound stimuli. We compared this statistical analysis with previously published data from classical psychophysical studies in loudness and pitch. Although speech sounds obviously do not include the full range of human auditory experience, they comprise both periodic (harmonic) and aperiodic (inharmonic) stimuli that are biologically important, strongly attended, and universally experienced. Moreover, previous analysis shows that the acoustic structure of speech is similar to that of a broad combination of environmental and animal sounds that make up the full complement of human auditory experience [14], suggesting that speech may have evolved to reflect this structure.

## Materials and Methods

### Speech Database

The previously recorded speech database consisted of calibrated full-bandwidth anechoic recordings (24-bit, 44.1-kHz sampling rate) of 20 semantically unpredictable phrases (for example “Amend the slower page”) [15]. The phrases were uttered by 15 speakers (8 female) at three intensity levels (soft, normal, and loud). Recordings were made with a Larson Davis 2551 precision microphone located 60 cm from the mouth at the level of the mouth.

### Determination of PDFs and CDFs

Probability distribution functions (PDFs) were calculated by counting the occurrences of different sound pressure level amplitude (*A*) or frequency (*F*) values (in 1-dB or 1-Hz wide bins) and dividing by the respective sum of all values. These values were used to obtain conditional PDFs (e.g., 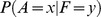) and marginal PDFs (e.g., 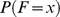) as reported in the Results section. CDFs (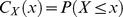) were obtained by computing the cumulative sum (integral) of the relevant PDF (see Introduction). We did not incorporate a model of peripheral auditory filtering because all of the psychophysical data to which the CDFs are compared were measured and reported in relation to the acoustic stimuli as such (i.e., distal to the auditory periphery).

#### Speech SPL distribution

The overall distribution of sound pressure levels (SPLs) in speech was determined using consecutive 20-ms segments extracted from the full speech corpus after excluding the silence between spoken passages. This distribution thus included both voiced and unvoiced speech. A 20-ms window length was chosen because changes in SPL over this period are minimal since 20 ms falls within the duration of brief phonemes, while remaining above the nominal threshold of auditory temporal resolution [16]. Using longer (50 ms) or shorter (10 ms) window lengths, however, did not significantly change the PDF obtained.

#### Harmonic tone frequency and level distributions

An autocorrelation algorithm [17] was used to detect voicing in a 50-ms window, which was advanced in consecutive 10-ms time steps of the speech corpus. Unvoiced speech frames were discarded. Fundamental frequency (F0) estimates were extracted for all 50-ms voiced speech segments with the algorithm (50 ms is the duration needed to include 3 cycles of a 60-Hz F0). The SPL at each F0 and its harmonics was obtained by calculating a 4096-point FFT for each 50-ms segment of voiced speech. A peak-picking algorithm was applied to each FFT spectrum that: 1) found the peak locations nearest the first three harmonic frequencies calculated from the autocorrelation F0 extraction; 2) calculated a new F0 estimate using the mean frequency difference between these peak locations; and 3) found the SPL peak nearest each new F0 estimate and its integer multiples up to 20,000 Hz. All harmonics at SPLs below the auditory threshold at that frequency [18] were excluded. The SPLs and frequency values (F0 and audible harmonics) were then used to calculate PDFs for the relevant psychophysical function as described in the Results section.

#### Comparison of the CDFs and psychophysical data

**Figure 1:**
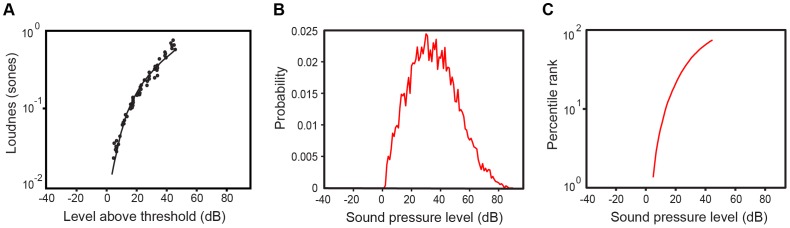
Qualitative comparison of pure tone loudness judgments with empirical predictions. (A) Loudness judgments of 1000-Hz pure tones below ∼45 dB SPL. The solid line is a best-fit curve to the psychophysical data obtained across nine different studies, redrawn from Buus et al [19]. (B) The probability distribution of SPLs of 1000-Hz tones extracted from the database. (C) The CDF derived from the data in (B) plotted for the same SPL range as in (A). The CDF follows the psychophysical function in (A).

**Figure 2:**
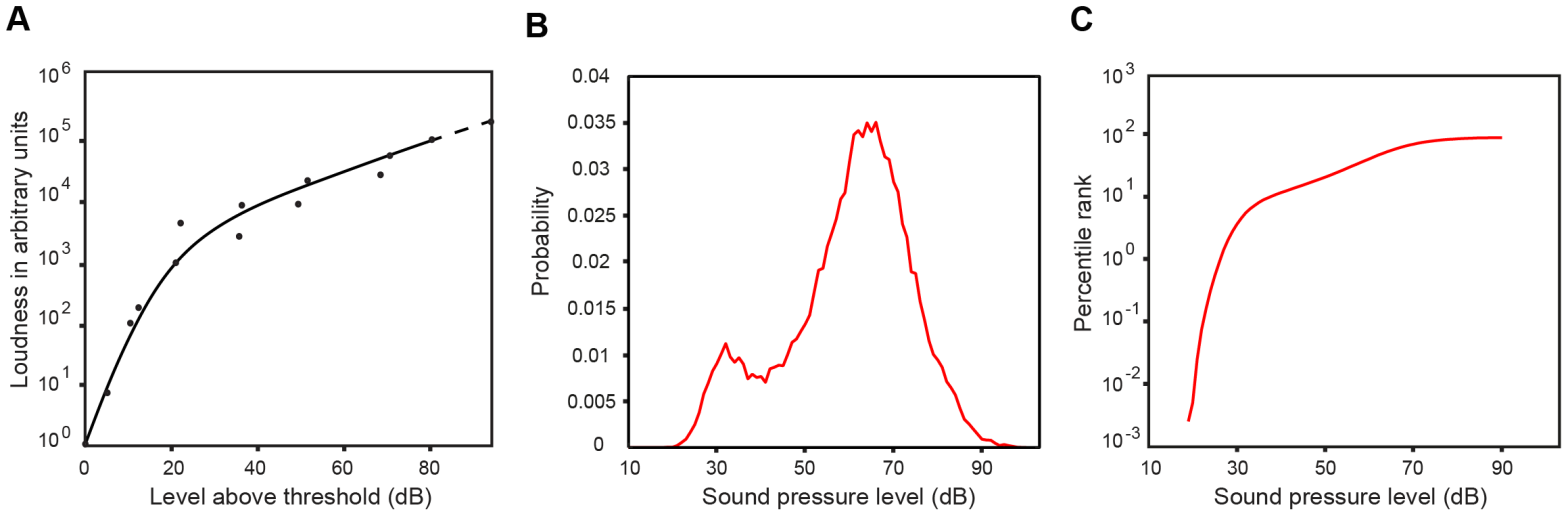
Qualitative comparison of speech loudness judgments with empirical predictions. (A) Speech loudness as a function of intensity (level above auditory threshold). The solid line represents the best-fit curve to the psychophysical data within the range of typical speech SPLs, redrawn from Fletcher and Galt [20]. The dashed line shows the trend beyond typical speech levels. (B) The probability distribution function of SPLs in the speech database. The bimodal shape is due to the characteristically different amplitudes of sustained speech sounds (i.e., vowels and some consonants) and transient speech sounds (e.g., aspiration noise from consonants). (C) The CDF derived from the PDF in (B) is generally similar to the psychophysical function in (A).

**Figure 3:**
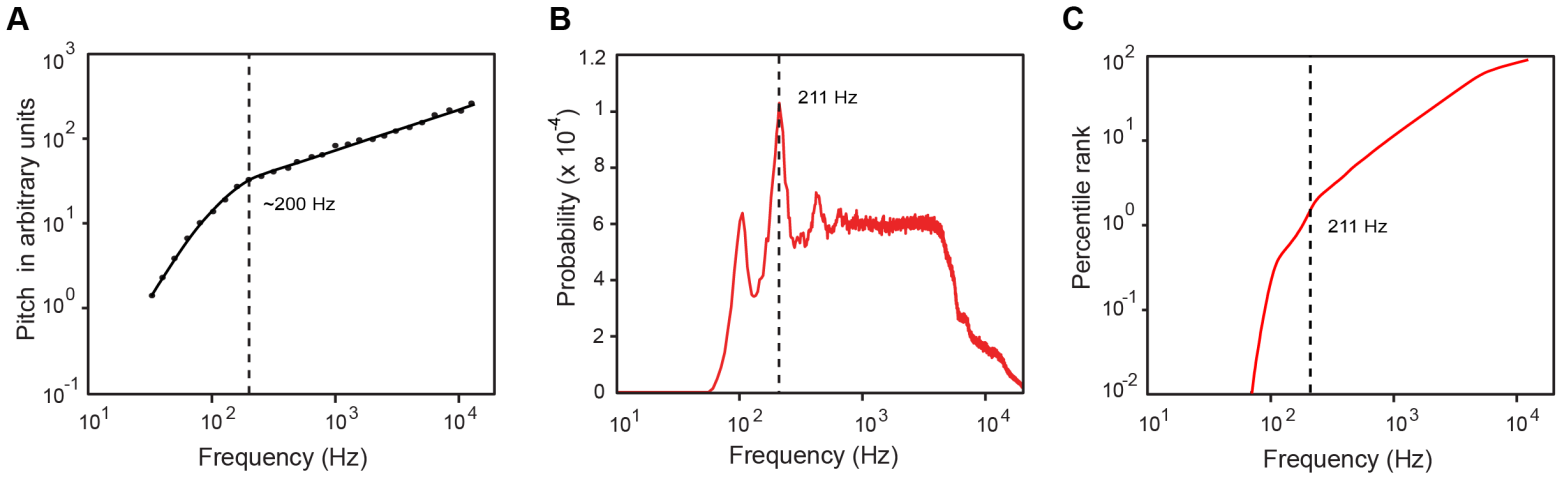
Qualitative comparison of pure tone pitch judgments with empirical predictions. (A) Judgments of pitch magnitude elicited by sine tones of increasing frequency, redrawn from Miskiewicz and Rakowski [23]. (B) The PDF of harmonic tones, obtained by taking integer multiples of fundamental frequencies in the database. (C) The CDF for harmonic tones in speech. As in (A), the CDF shows a distinct change in slope at ∼200 Hz.

**Figure 4:**
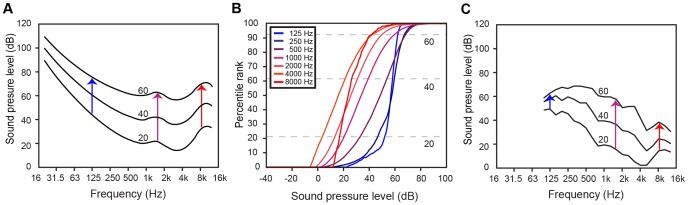
Qualitative comparison of the rate of growth in loudness as a function of frequency with empirical predictions. (A) Equal loudness contours for 20, 40, and 60 dB SPL at 1 kHz, calculated from the ISO standard [18]. The rate of loudness growth (arrows) is greater for low and very high frequencies than for middle frequencies. (B) The CDFs for SPLs of individual harmonic tones, derived from the database for standard octave frequencies. The slopes of the CDFs decrease as frequency increases to ∼4 kHz, and then increase again at higher frequencies. Dotted lines indicate the percentile ranks for 20, 40 and 60 dB on the 1-kHz tone CDF. (C) Empirically predicted “equal percentile rank” contours taken from CDFs in (B). Contours were plotted as the SPL values from the CDFs (obtained for standard 1/3–octave frequencies) that had the same percentile rank as 20, 40, and 60 dB on the 1-kHz CDF. The rate of loudness growth as a function of frequency is similar to that in (A).

**Figure 5:**
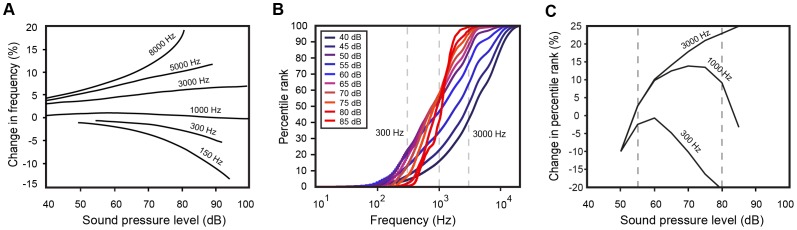
Qualitative comparison of pitch judgments as a function of intensity with empirical predictions. (A) The effect of intensity on perceived frequency, redrawn from Houtsma [29] and Stevens [26]. (B) The relationship between harmonic tones and SPLs. The percentile ranks of low-frequency tones (e.g. 300 Hz) decrease as intensity increases, while the reverse occurs for high-frequency tones (e.g. 3000 Hz). (C) The empirically predicted effect of intensity on three frequencies (300, 1000, and 3000 Hz) taken from the CDFs of different SPL values. To facilitate comparison with (A), the data are re-plotted as changes in percentile rank for a given frequency as intensity increases.

The published psychophysical data for loudness are often reported as a function of level above threshold. The CDFs were therefore plotted over comparable ranges based on published thresholds for the given stimulus (see Figures 1C, 2C, 3C, 4B and 5B). Absolute intensity depends on distance; the speech in the database was recorded at 60 cm, which is a normal speaker distance.

## Results

The following sections consider simple psychophysical functions that directly relate loudness to intensity, and pitch to frequency. We then examine compound perceptual phenomena in which varying one physical parameter (intensity or frequency) affects perception of the other. In each figure we first plot the data from the classical psychophysical literature, followed by the relevant empirical analysis of the database for comparison.

### Simple Loudness and Pitch Functions

Standard pure tone loudness judgments are typically obtained using 1-kHz sine wave stimuli. To compare psychophysical judgments obtained in this way with predictions from the empirical approach, we determined a conditional PDF of intensities by extracting the SPL at 1 kHz for each occurrence of a 1-kHz harmonic tone in voiced speech (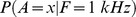). Since the amplitudes of voiced speech at 1 kHz (i.e., the harmonics of fundamental frequencies in the normal range of ∼70–400 Hz; see Fig. S2) have relatively low SPL values, the relevant comparison to the psychophysical data is limited to stimuli below ∼45 dB SPL. Psychophysical judgments of pure tone loudness over this range [19] are shown in Fig. 1A, and the PDF of harmonic tone intensities in speech at 1 kHz in Fig. 1B. Figure 1C shows that the CDF of the data in Fig. 1B is in close agreement with the low-amplitude psychophysical function in Fig. 1A (see also Fig. S4A).

Some of the earliest studies of loudness specifically examined responses to speech sound stimuli. Figure 2A shows that the classical speech loudness function follows a power law that is steeper at lower intensities [20], [21]. Figure 2B shows the PDF of full-bandwidth SPLs calculated for consecutive 20-ms intervals of speech in the database (see Methods). The CDF in Fig. 2C shows similarities to the psychophysical data.

While the reasons for variation in reports of pitch magnitude scaling are debated, the classic studies show that judgments of pitch magnitude for pure tones increase linearly above ∼200 Hz on a log-log scale as frequency increases [3], [22], [23]. The most recent data available show that the slope is steeper, however, at frequencies below 200 Hz [23] (Fig. 3A). To compare these observations with empirical predictions, we calculated the marginal PDF of all occurrences of fundamental frequencies (F0s) and their higher harmonics in voiced speech in the database, 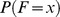 (Fig. 3B). Figure 3C shows the resulting CDF for experience with harmonics in voiced speech. The CDF is once again qualitatively similar to the psychophysical data (see also Fig. S4B), but the slopes are quantitatively different. Whereas the psychophysical data above and below 200 Hz follow power laws with slopes 1.74 and 0.478, respectively, the corresponding CDF slopes are 2.3 and 1, respectively.

### Compound Loudness and Pitch Functions

Equal loudness contours spanning the audible frequency spectrum are well documented in human psychophysics [18], [24], [25]. The contours in Fig. 4A show the classic psychophysical model data for intensity levels of pure tone frequencies that are perceived to be equally loud. The salient feature of these contours is that the rate of growth of loudness with intensity (i.e., the distance between equal loudness contour lines) varies with frequency. Lower and very high frequencies show greater rates of loudness growth (less distance between the contours) than do middle frequencies. If this phenomenon is based on experience with acoustic stimuli, then conditional CDFs obtained for SPL values of individual frequencies (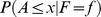, where *f* is a specified frequency) should show corresponding differences in the rate of loudness growth. The rate should be greater for a CDF obtained for a low or very high frequency than for a middle frequency (see Text S1 and Fig. S3A for further explanation).

Figure 4B shows the conditional CDFs for the SPL values of harmonic tones obtained from the database at standard octave frequencies. These curves were generated by calculating the SPL CDFs of all harmonics falling within frequency bins centered at third-octave band frequencies. (For frequencies less than 250 Hz, a 10-Hz wide bin was used; for frequencies between 250–500 Hz, a 20-Hz wide bin was used; and for frequencies greater than 500 Hz, a 40-Hz wide bin was used.) The conditional PDFs for the individual frequencies are not shown, but were roughly similar in shape to the PDF for the 1-kHz harmonics in Fig. 1B. The equal loudness curves predicted by equivalent percentile ranks on the conditional CDFs are plotted in Fig. 4C. These “equal rank” contours were generated by determining the percentile ranks of 20, 40 and 60 dB on the 1-kHz CDF, and plotting SPL values with equivalent percentile ranks for all other frequencies. The rate of loudness growth (i.e., the variation in distance between the empirically predicted curves) shows the same qualitative trend as the psychophysical curves in Fig. 4A, although again there are quantitative discrepancies. Specifically, the difference in loudness growth rate between the observed and predicted data at low and high frequencies is quite large. At 125 Hz, for example, the equal loudness contours show a 32-dB change on the ordinate between the upper and lower curves shown, while the empirical approach predicts only a 15-dB change. Similarly, at 8 kHz the psychophysical curves show a 38-dB change, while the prediction is a 21-dB change.

The effect of intensity on the pitch of pure tone frequencies has also been the focus of several psychophysical studies [26]–[29]. Although the effect is small, increasing intensity typically: 1) decreases pitch for frequencies below ∼1000 Hz; 2) has little or no effect on pitch for frequencies between 1000–2000 Hz; and 3) increases pitch for frequencies above ∼2000 Hz [29] (Fig. 5A). If these phenomena are based on experience, the percentile rank of a low-frequency tone (e.g., 300 Hz) should decrease when intensity increases, and vice versa for a high frequency (e.g., 3000 Hz). We examined this prediction by comparing conditional CDFs for harmonic tones at individual intensities (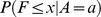, where *a* is a specified intensity amplitude). The implication is that stimulus intensity provides a context that alters judgments of pitch (see Text S1 and Fig. S3B for further explanation).

Figure 5B shows the conditional CDFs for harmonic tones extracted from the speech database and separated by their associated SPL values. These curves were generated by calculating the conditional CDFs for all harmonic frequencies separated by SPL in 5-dB-wide bins centered at a given SPL (see Fig. 5B). The curves follow the predicted trend over a small range of levels (∼55–80 dB), but not over the entire range. To facilitate comparison with the classic psychophysical observations in Fig. 5A, the data in Fig. 5B are re-plotted in Fig. 5C as changes in percentile rank with increasing intensity. Once again, the qualitative trend is similar to the published data for perceived pitch changes, but only over a limited SPL range and with large quantitative discrepancies. For example, the curves for both 300 and 3000 Hz predict pitch shifts up to 20 percentile points or more with increasing intensity. If these percentile changes are converted to percentage change from a reference percentile at 50 dB SPL, the predicted shifts amount to a 60% increase at high frequencies and a 100% decrease at low frequencies. These values are much larger than those reported in the psychophysics literature, which typically fall between 2–4%.

## Discussion

We examined classic loudness and pitch psychophysical functions, asking whether the observations accord with predictions made on the basis of the frequency of occurrence of intensities and frequencies in a representative subset of natural acoustic stimuli. In each instance, the psychophysical functions are qualitatively similar to the CDFs obtained from the database, although quantitative differences are apparent in most cases (see Text S1 for statistical evaluation; see also Fig. S1).

Accordingly, these correlational comparisons must be viewed with caution. To complicate the interpretation further, nearly all previous psychophysical data on loudness and pitch have been reported on log-log scales, making comparison with CDFs (which are bounded percentile scales) indirect. Nonetheless, the observations are consistent with an empirical strategy of audition, and are difficult to explain away. The qualitative agreement between the observed and predicted functions for loudness and pitch suggests that what we hear may be determined not by the physical parameters of a stimulus *per se*, but by its cumulative frequency of occurrence (percentile rank) within the range of human experience with similar stimuli. Since the neural circuitry underlying percepts of intensity and frequency would presumably reflect this representation of acoustic stimuli, the empirical approach could be useful in relating neural responses to perception.

At the least, an experience-based concept of audition offers a plausible rationale for otherwise puzzling phenomena. For example, the perceived loudness of two speech sounds with SPLs of 50 and 56 dB fall at the 38^th^ and 54^th^ percentile, respectively, on the CDF in Fig. 2C. (Note that a percentile does not indicate the percentage of the time a stimulus is heard, but rather the percentage of stimuli that are lower in SPL that the given stimulus.) The difference of 6 dB is a doubling of sound pressure and might thus be expected to cause a doubling of loudness. In the accumulated experience with such stimuli, however, the more intense stimulus has typically been less than twice as great on a percentile scale (∼1.4 times as great), in accord with loudness psychophysics. At lower intensities the CDF for speech SPLs is steeper because of the peak in the frequency of occurrence of low-amplitude speech sounds (see the PDF in Fig. 2B), again in agreement with the psychophysical data. The nonlinearity of the loudness function at low intensity levels (see Fig. 1) and the frequency-dependence of loudness growth (see Fig. 4) can also be rationalized by the occurrence of individual harmonic tones at different intensities in natural sounds.

Similarly, an empirical framework could offer a straightforward account for pitch phenomenology in terms of routine human experience with occurrences of tones, of which the most prominent natural sources are the F0s and higher harmonics in voiced speech. The harmonic tone PDF peak at ∼200 Hz results from the most common female F0 coinciding with the second harmonic of the most common male F0 (see Fig. 3B and Fig. 2). The sharp decrease and flatter distribution of harmonic tone incidences at higher frequencies in the PDF causes a bend in the CDF at about 200 Hz (see Fig. 3C), thus predicting a shift in perception of frequency above this value (see Text S1). The results in Fig. 5 show that the intensity-dependence of pitch is also roughly predicted by harmonic tones occurring at individual intensities. Several other aspects of pitch elicited by complex tones have similarly been explained by accumulated experience with patterns of harmonic tones in speech [30].

### Limitations of an Empirical Analysis

While speech provides a reasonable database of commonly experienced sound stimuli, it is an incomplete representation of natural acoustic stimuli for humans. For example, we could not assess the standard loudness function in Fig. 1A at intensities above ∼45 dB because ∼75% of 1-kHz tones in speech are below this level (see Text S1). It is also obvious that the database used here does not account for everyday experience with conversational speech at differing distances. Including such experience could affect the analysis, as indicated in Fig. S5. Another significant limitation is the inability of the approach used in this report to predict perception of novel acoustic patterns. Finally, except for speech loudness, the predictions had to be compared with psychophysical functions determined using pure tones, the conventional experimental approach in auditory psychophysics. Pure tones are rarely if ever part of natural human experience, and we thus had to assume that the way pure tones are heard is based on the evolution of responses to tones in naturally occurring stimuli. Although voiced speech is the prevalent source of such stimuli in human experience, the auditory system is unable to resolve harmonics from a broadband stimulus when multiple harmonics fall within a single auditory filter band [31]. Thus harmonics of complex tones are not readily perceived as individual pitches, but contribute to the perception of the fundamental frequency of complex stimuli. Furthermore, harmonic tone extraction does not account for partial loudness, loudness summation, or any masking of pure tones within broadband stimuli. The idea that we derive our subjective sense of pure tone loudness or pitch from harmonics in voiced speech is based primarily on the absence of any obvious alternative. All of these issues may contribute to the imprecision of the comparisons we report.

### Standard Explanations of Loudness and Pitch

#### Loudness

Loudness is generally explained in terms of spectral amplitudes of sound stimuli, how the spectrum is modulated by the transfer functions of the peripheral auditory system, and its eventual translation into the firing rates of auditory neurons [4], [32]–[34]. Consistent with this interpretation, some psychophysical observations are predicted by phenomenological models based on spectral representations of sound stimuli filtered through physiologically derived transfer functions [35], [36].

The standard model is described in the ANSI standard for loudness calculation [36], [37]. Although this model generates good approximations of equal loudness curves, pure tone loudness functions, loudness summation of white noise, and loudness matching of a pure tone in quiet and noise, it entails many assumptions about the nature of basilar membrane mechanics and neural signaling [36], and there are many psychophysical observations that it does not predict [34]. Some physiological evidence also contradicts the idea of filtering by the peripheral auditory system as a basis for the observed psychophysical data. In experimental animals, for instance, measurements of neural spike rates in the auditory nerve fail to account for loudness psychophysics [38]. In short, the current understanding of loudness appears incomplete, and an empirical approach may be relevant to understanding the genesis of this perceptual quality. This conception of audition makes no assumption about basilar membrane motion or filtering, and, in fact, may provide insight into why cochlear evolution has led to its present day structure and acoustical response.

#### Pitch

The phenomenology of pitch is typically explained in terms of a frequency-encoding “place” theory based on the location of basilar membrane vibration, or a “temporal” theory based on neuron spiking intervals and phase-locking. The relative merits of these two theories have long been debated, and while each accords with some aspects of pitch psychophysics, they fail to explain others [39], [40].

For example, proponents of the place theory of pitch have argued that the pitch-versus-intensity effect shown in Fig. 4A discredits the temporal theory, attributing the effect instead to shifts in the location of maximal basilar membrane vibration observed in the mammalian cochlea [41], [42]. Other work, however, has shown that neither direct measurements of shifts in basilar membrane deflection in experimental animals [43] nor indirect measurements in humans [44] accord with observed psychophysical shifts. As a result, there is no generally accepted explanation for changes in pitch as a function of intensity [40]. Moreover, neither theory accounts for the pitch function shown in Fig. 3A. The change in slope of the function at ∼200 Hz has simply been attributed to the diversity of neurophysiological mechanisms of pitch encoding for the frequency ranges below and above 200 Hz [23]. Here again an empirical perspective may help clarify the current understanding of pitch.

### Why an Empirical Framework is Plausible

It has long been clear in vision that many aspects of what we see are incommensurate with the physical characteristics of retinal stimuli; in this sensory modality, psychophysical observations seem best explained by an experience-based strategy that evolved to deal with the inverse optics problem [7], [8]. The present work suggests that audition may operate on this same basis for the same general reasons. Both vision and audition must contend with the fact that sensory stimuli received by biological sensors inevitably conflate the many physical parameters of real-world sources that must in some sense be apprehended for behavior to be successful (see Introduction). A strategy of empirically ranking according to the frequency of occurrence of natural stimulus patterns may allow behavior to succeed despite the conflation of the generative sources in sensory stimuli (op cit.).

While some studies have explored how the statistics of natural sounds are represented in the auditory system [14], [45]–[47], and others have examined how context affects learning [48], [49], as far as we are aware no one has proposed that audition operates on the empirical basis (percentile ranking) presented here. If this hypothesis of auditory perception is correct, auditory circuitry will eventually need to be understood within this framework.

## Supporting Information

Figure S1 **Empirical analysis for speech loudness and pitch using the TIMIT database.** (A) The PDF of SPLs taken from 20-ms frames of the TIMIT database. Similar to the main results, the CDF shows a bimodal distribution representing levels from vowels and consonants. (B) The CDF calculated from data in (A). Similar to the results reported, the CDF is steeper at lower intensities. (C) The PDF of pure tone frequencies (F0s and harmonics) taken from the TIMIT database. As in the text, the PDF shows a peak at ∼200 Hz with flatter distribution beyond this frequency. (D) The CDF calculated from data in (C). As in the text, the CDF shows generally linear behavior with a distinct change at ∼200 Hz.
(TIF)

Figure S2 **Probability distribution of fundamental frequencies in speech.** As expected, the PDF is a bimodal distribution with peaks at typical male (106 Hz) and female (209 Hz) F0s.
(TIF)

Figure S3 **Empirical interpretation of compound auditory phenomena.** (A) Ideal empirical interpretation of loudness judgments as a function of frequency. The rate of growth (slope) of the CDF is greater for low and high frequencies than for middle frequencies. (B) Ideal empirical interpretation of pitch judgments as a function of intensity. The percentile ranks of low frequencies decrease as intensity increases, while ranks of high frequencies increase.
(TIF)

Figure S4 **Correlation of psychophysical and empirical data.** (A) 1-kHz loudness function and 1-kHz CDF. The percentile rank can account for ∼98% of the low-amplitude loudness data (*R* ^2^ = 0.984). (B) Pure tone pitch function and harmonic tone CDF. The percentile rank can account for ∼96% of the pure tone pitch data (*R* ^2^ = 0.962).
(TIF)

Figure S5 **Loudness beyond the range of the speech database.** (A) Loudness of a 1-kHz tone. The 1-kHz CDF (red) follows the low-amplitude psychophysical data, but does not predict the loudness function above ∼45 dB SPL. The solid black line shows the traditional loudness curve above 45 dB SPL (slope = 0.3). The dotted black line shows a revised loudness function combining the low-amplitude function with a loudness function having a slope of 0.19 at moderate levels and 0.4 at high levels (adapted from Buus et al., 1998, and Florentine et al., 1996). (B) Same as (A) but with the 1-kHz CDF including self-produced 1-kHz harmonic tones. In this case, the CDF predicts loudness up to ∼60 dB SPL.
(TIF)

Text S1 (PDF)
