# Supplementary figures and images for: Are Auditory Percepts Determined by Experience?
Source: PLoS One. 2013 May 7;8(5):e63728. doi: 10.1371/journal.pone.0063728 (PMC3646789; doi:10.1371/journal.pone.0063728)

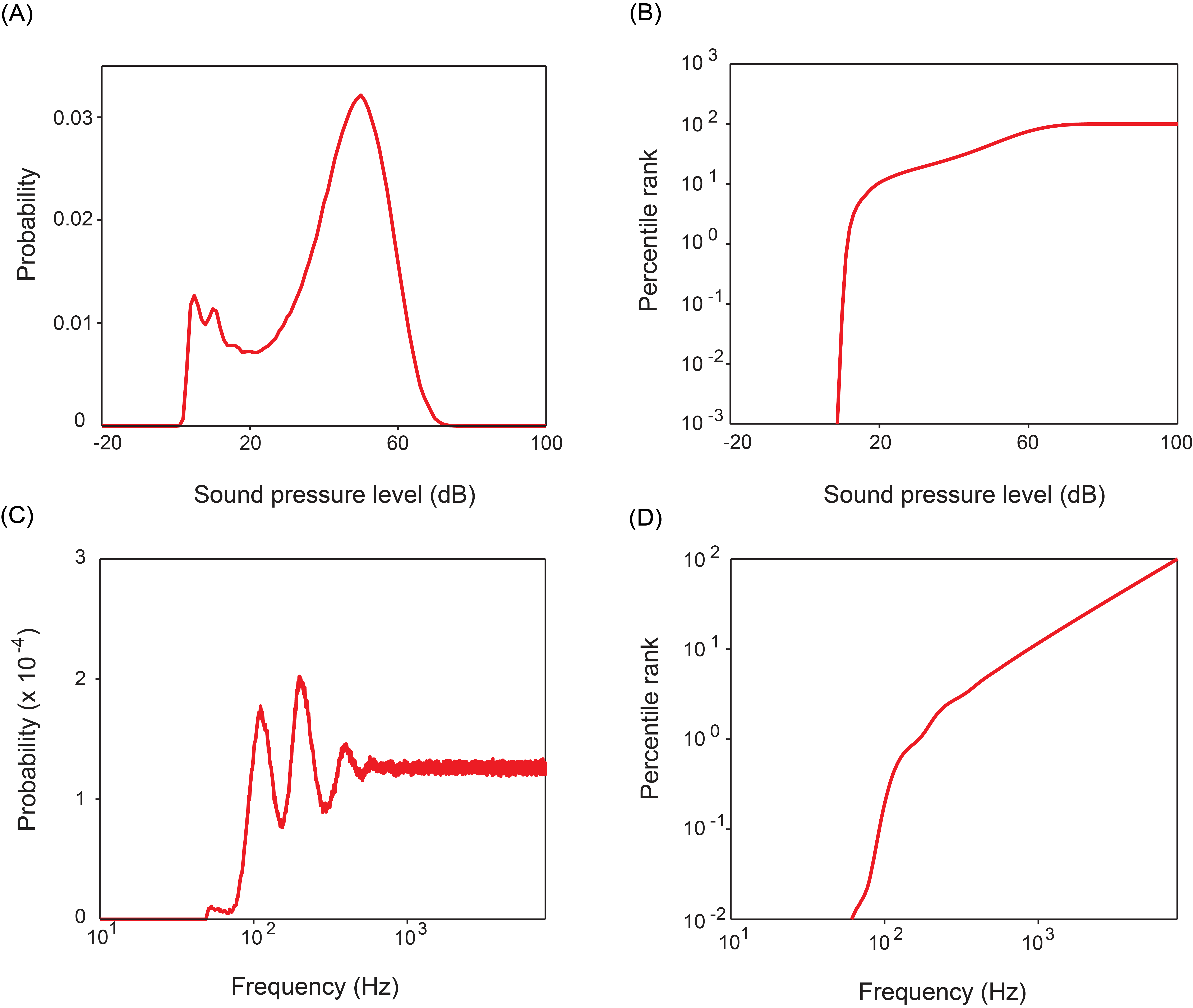

Supplement: Figure S1 — Empirical analysis for speech loudness and pitch using the TIMIT database. (A) The PDF of SPLs taken from 20-ms frames of the TIMIT database. Similar to the main results, the CDF shows a bimodal distribution representing levels from vowels and consonants. (B) The CDF calculated from data in (A). Similar to the results reported, the CDF is steeper at lower intensities. (C) The PDF of pure tone frequencies (F0s and harmonics) taken from the TIMIT database. As in the text, the PDF shows a peak at ∼200 Hz with flatter distribution beyond this frequency. (D) The CDF calculated from data in (C). As in the text, the CDF shows generally linear behavior with a distinct change at ∼200 Hz. (TIF) [file pone.0063728.s001.tif]

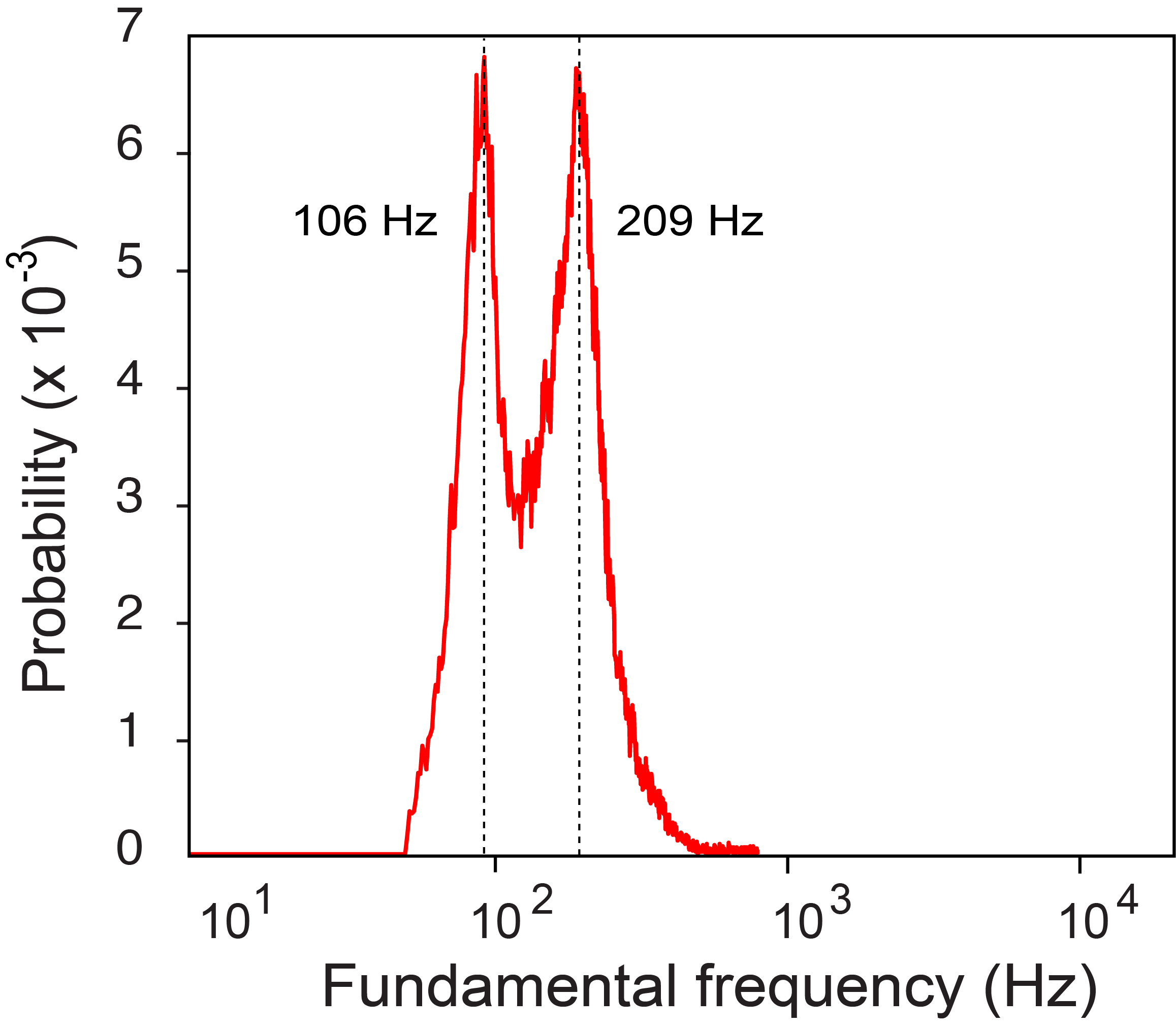

Supplement: Figure S2 — Probability distribution of fundamental frequencies in speech. As expected, the PDF is a bimodal distribution with peaks at typical male (106 Hz) and female (209 Hz) F0s. (TIF) [file pone.0063728.s002.tif]

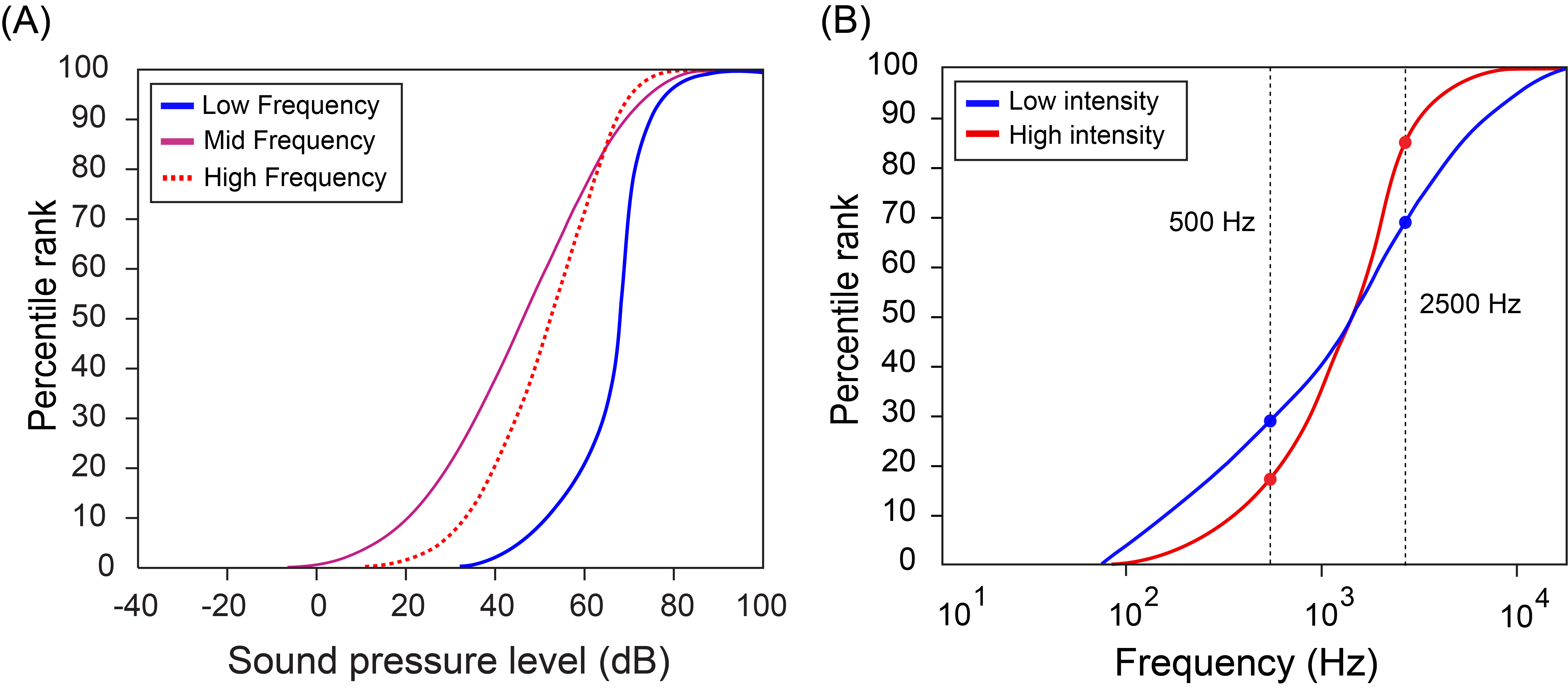

Supplement: Figure S3 — Empirical interpretation of compound auditory phenomena. (A) Ideal empirical interpretation of loudness judgments as a function of frequency. The rate of growth (slope) of the CDF is greater for low and high frequencies than for middle frequencies. (B) Ideal empirical interpretation of pitch judgments as a function of intensity. The percentile ranks of low frequencies decrease as intensity increases, while ranks of high frequencies increase. (TIF) [file pone.0063728.s003.tif]

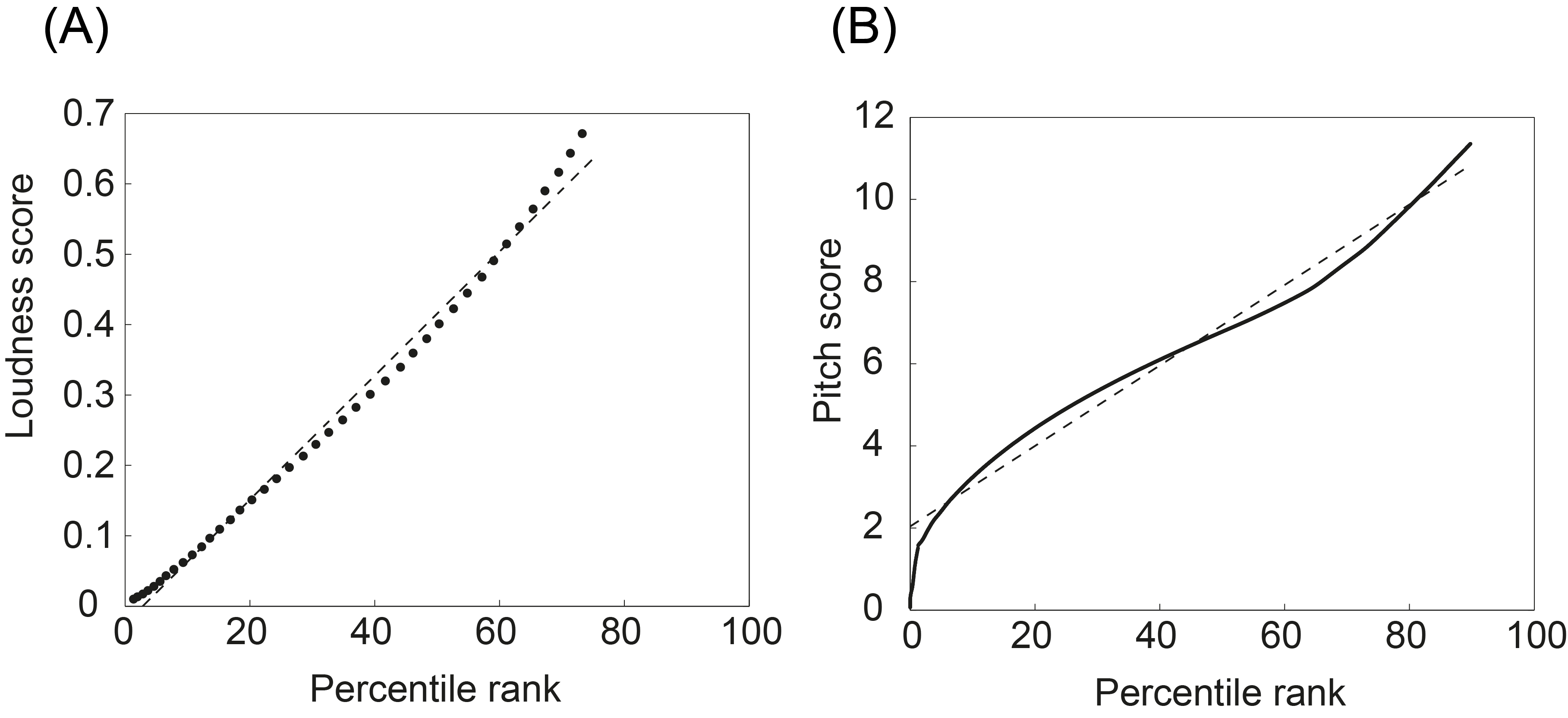

Supplement: Figure S4 — Correlation of psychophysical and empirical data. (A) 1-kHz loudness function and 1-kHz CDF. The percentile rank can account for ∼98% of the low-amplitude loudness data (R 2 = 0.984). (B) Pure tone pitch function and harmonic tone CDF. The percentile rank can account for ∼96% of the pure tone pitch data (R 2 = 0.962). (TIF) [file pone.0063728.s004.tif]

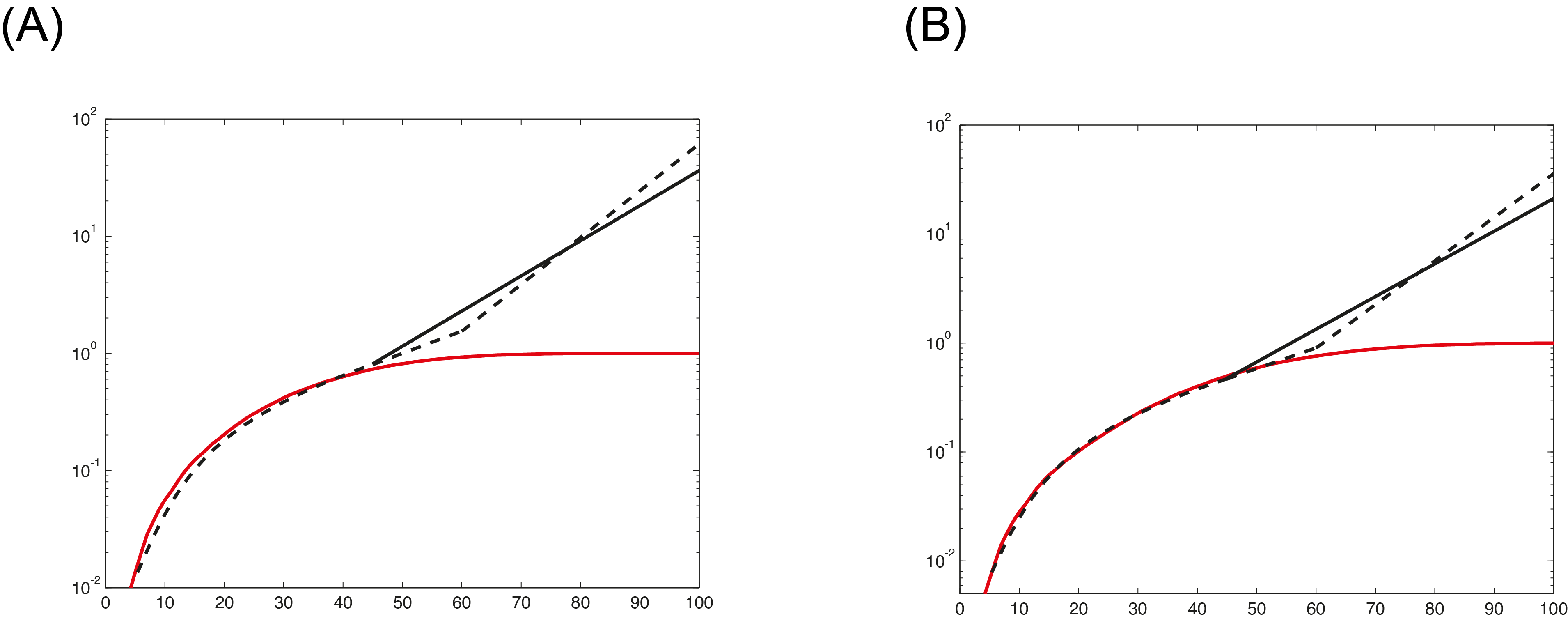

Supplement: Figure S5 — Loudness beyond the range of the speech database. (A) Loudness of a 1-kHz tone. The 1-kHz CDF (red) follows the low-amplitude psychophysical data, but does not predict the loudness function above ∼45 dB SPL. The solid black line shows the traditional loudness curve above 45 dB SPL (slope = 0.3). The dotted black line shows a revised loudness function combining the low-amplitude function with a loudness function having a slope of 0.19 at moderate levels and 0.4 at high levels (adapted from Buus et al., 1998, and Florentine et al., 1996). (B) Same as (A) but with the 1-kHz CDF including self-produced 1-kHz harmonic tones. In this case, the CDF predicts loudness up to ∼60 dB SPL. (TIF) [file pone.0063728.s005.tif]
