# Supplementary material for: Are Auditory Percepts Determined by Experience?
Source: PLoS One. 2013 May 7;8(5):e63728. doi: 10.1371/journal.pone.0063728 (PMC3646789; doi:10.1371/journal.pone.0063728)
Supplement: Text S1 — (PDF) [file pone.0063728.s006.pdf]

SI Text

Results

To confirm our results with another database, we used a subset of the TIMIT speech corpus [50], which includes 10 sentences spoken by 384 speakers (192 female) from different American English dialects. The analysis was the same as described in the text. While the quality of the TIMIT recordings precludes accurate determination of harmonic tone SPLs, similar trends were observed for the analysis of speech loudness and pure tone pitch (Fig. S1).

Discussion

Distribution of frequencies in speech

Figure S2 shows the PDF of F0s in the database we used. In accord with previous work, the PDF is bimodal with approximately half of the data points distributed around ~100 Hz and the other half distributed around ~200 Hz, representing the characteristic speaking F0s of males and females, respectively.

The PDF of harmonic tones is characterized by a peak at 211 Hz and a relatively even distribution of high frequencies (Fig. 3B). The peak at 211 Hz is a result of the most common female F0 (209 Hz), together with the second harmonic of the most common male F0 ($2\times106 = 212$ Hz). Another peak at ~400 Hz similarly arises as the combination of the second harmonic of the female F0 and fourth harmonic of the male F0. The absence of distinct harmonic peaks thereafter is due to the wide distribution of F0s with slightly different values. Since small differences in F0s result in harmonics with larger differences, the precise overlap of frequencies declines rapidly as frequency increases,

resulting in the flat distribution out to ~5 kHz seen in Fig. 3B. Above ~5 kHz the characteristic decrease in speech energy amplitude and simultaneous increase in auditory intensity thresholds leads to a decrease in the PDF as many higher harmonics in this range fall below auditory threshold. This change in the PDF at 5 kHz has only a slight effect on the CDF (see Fig. 3C), but does, on empirical grounds, predict some change in perception of frequency above 5 kHz. This observation accords with observed changes in pitch psychophysics above 5 kHz [51, 52].

Empirical prediction of pitch versus intensity and equal loudness curves

Figure S3A depicts the expected (theoretical) relationship of loudness judgments for low, middle, and very high frequencies based on an empirical interpretation of the equal loudness curves shown in Fig. 4A. The slopes of the curves are greater for low (and very high) frequencies than for middle frequencies, in conformance with psychophysical data on loudness growth. The data collected from the speech database follows this expectation (see Fig. 4B). This result is similar to a context effect, in which frequency serves as the context for loudness.

Figure S3B depicts the expected relationship of pitch judgments for high and low intensities based on an empirical interpretation of the psychophysical data shown in Fig. 5A. The percentile rank of low frequencies (e.g. 500 Hz) decreases as intensity increases, while the reverse is observed for high frequencies (e.g. 2500 Hz). The crossover point between the two curves that does not change empirical rank is ~1 kHz. The data collected from the speech database again follows the empirical expectation (see Fig. 5B). This

observation is similar to a lightness contrast effect in vision, intensity serving as the context for pitch.

Fitting psychophysical data to the CDF

Plotting the 1-kHz loudness scores (calculated from the equation given by Buus et al.) against the percentile rank values on the 1-kHz loudness CDF shows strong correlation between the psychophysical function and the CDF (Fig. S4A). Plotting the pitch scores (calculated from the power law parameters given by Miskiewicz and Rakowski) against the percentile rank values on the pitch CDF shows strong correlation also, though this is largely due to the data above 200 Hz (Fig. S4B). While the slope values of the psychophysical and empirical pitch functions are not identical, it should be noted that the slope of the psychophysical pitch scale varies with the method used [22]. Neither raw data nor best-fit equations for the nonlinear speech loudness function are available, precluding statistical analysis for this function. Some have published power law exponent values for speech loudness data (e.g., [53]), but these studies approximate the data with a single straight line on log-log scale, whereas the data (both here and in previous studies) show a concatenation of multiple power law relationships.

Limitations of the auditory environment

The empirical prediction of the 1-kHz loudness function does not accurately predict loudness above ~45 dB SPL since only ~25% of occurrences of 1-kHz tones in speech are above 45 dB SPL. Above 45 dB SPL, the standard loudness function increases as a power law with an exponent of ~0.3, but the empirical data increases asymptotically to a saturation value of 100% (Fig. S5A). Similarly, the empirical prediction of the speech

loudness function does not accurately predict loudness above 90 dB SPL since less than 0.05% of sounds in the database were above 90 dB SPL.

It is possible that a more accurate representation of human auditory experience would resolve this discrepancy. For example, analysis of the speech database does not account for occurrences of 1-kHz harmonics in self-generated speech, which are audible and ~22 dB higher than the 1-kHz harmonics in speech heard at 60 cm [54]. The CDF shown in Fig. S5B results from the assumption that 50% of 1-kHz harmonics heard by humans are self-produced. The CDF follows the dotted curve (adapted from Buus et al. [19] and Florentine et al. [55]) up to ~60 dB. Further changes to the CDF may result from other sources of 1-kHz tones (infant cries, non-human animal vocalizations, etc.); it is unlikely, however, that any natural sounds produce 1-kHz tones above 100 dB SPL. An empirical prediction for perception of intensities beyond the range of human experience is not possible.

References

50. Garofolo, J. S., Lamel, L. F., Fisher, W. M., Fiscus, J. G., Pallett, D. S., and Dahlgren, N. L. (1990). DARPA-TIMIT Acoustic-phonetic continuous speech corpus [CD-ROM]. US Department of Commerce, Gaithersburg, MD.

51. Moore, B. C. J. (1973). Frequency difference limens for short-duration tones. J Acoust Soc Am 54:610-619.

52. Oxenham, A. J., Micheyl, C., Keebler, M. V., Loper, A., and Santurette, S. (2011). Pitch perception beyond the traditional existence region of pitch. Proc Natl Acad Sci 108(18):7629-7634.

53. Mendel, M. I., Sussman, H. M., Merson, R. M., Naeser, M. A., and Minifie, F. D. (1969). Loudness judgments of speech and nonspeech stimuli. J Acoust Soc Am 46(6):1556-1561.

54. Reinfeldt, S., Ostli, P., Hakansson, B., and Stenfelt, S. (2010). Hearing one's own voice during phoneme vocalization—Transmission by air and bone conduction. J Acoust Soc Am 128:751-762.

55. Florentine, M., Buus, S., and Poulsen, T. (1996). Temporal integration of loudness as a function of level. J Acoust Soc Am 99(3):1633-1644.
